# Supplementary material for: Efficient electrocatalytic acetylene semihydrogenation by electron–rich metal sites in N–heterocyclic carbene metal complexes
Source: Nat Commun. 2021 Nov 12;12:6574. doi: 10.1038/s41467-021-26853-0 (PMC8589958; doi:10.1038/s41467-021-26853-0)
Supplement: Supplementary file 1 — Supplementary Information [file 41467_2021_26853_MOESM1_ESM.pdf]

Supporting Information

Efficient electrocatalytic acetylene semihydrogenation by electron–rich metal sites in N–heterocyclic carbene metal complexes

Lei Zhang, [1] *Zhe Chen,*[2] *Zhenpeng Liu,*[1] *Jun Bu,*[1] *Wenxiu Ma,* [1] *Chen Yan,* [1] *Rui Bai,* [1] *Jin Lin,* [1] *Qiuyu Zhang,*[1] *Junzhi Liu,*[3] *Tao Wang*[2,*] *and Jian Zhang*[1,*]

[1] Key Laboratory of Special Functional and Smart Polymer Materials of Ministry of Industry and Information Technology and Department of Advanced Chemical Engineering, School of Chemistry and Chemical Engineering, Northwestern Polytechnical University, Xi'an 710129, P. R. China.

[2] Center of Artificial Photosynthesis for Solar Fuels, School of Science, Westlake University, Hangzhou 310024, China.

[3] Department of Chemistry and State Key Laboratory of Synthetic Chemistry, The University of Hong Kong, Pokfulam Road, Hong Kong, China.

Emails: twang@westlake.edu.cn; zhangjian@nwpu.edu.cn

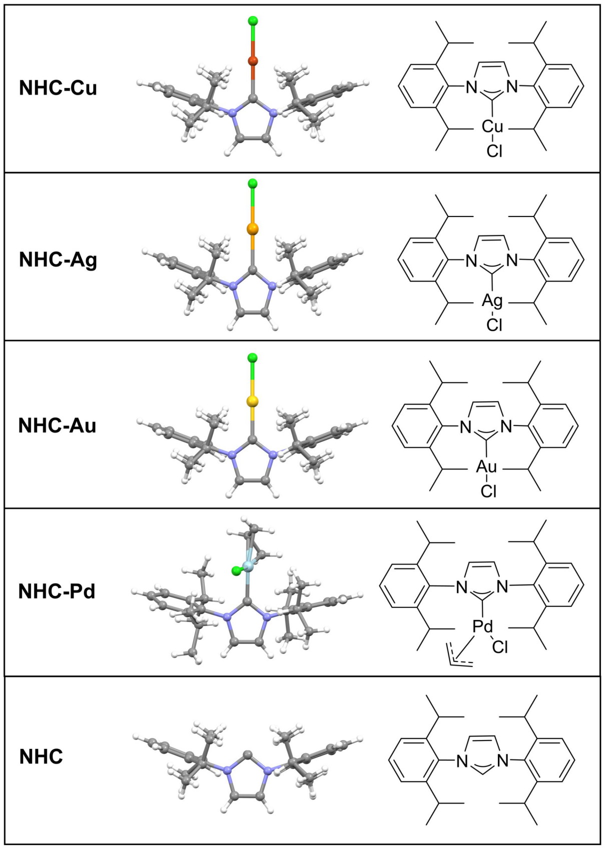

Supplementary Figure 1 | Molecular structures of NHC–metal complexes. The red, orange, yellow, blue, green, violet, gray and white spheres represent Cu, Ag, Au, Pd, Cl, N, C and H, respectively.

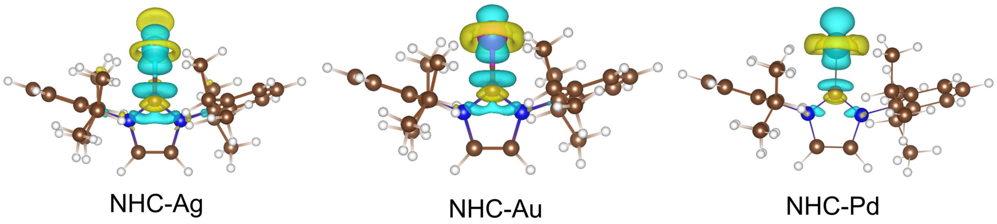

Supplementary Figure 2 | Charge distributions of NHC with different metal atoms, where the isosurface value is set to be 0.004 e Å^{-3} and the charge accumulated and depleted regions are shown in yellow and cyan, respectively.

Supplementary Table 1 | The calculated adsorption free energy (G_H, eV) of H (H^*) at metal sites of NHC–metal complexes.

Metal site	Cu	Ag	Au	Pd
ΔG_H	–1.13	–0.82	–1.23	0.51

Supplementary Table 2 | The calculated total energy (E_{tot}), zero–point energy (E_{ZPE}) and entropic (E_S) at 298.15 K of gaseous H_2, CHCH, CH_2CH_2 and CH_3CH_3 (the unit is eV).

Gas species	E_{tot}	E_{ZPE}	E_S
H_2	–6.978	0.285	0.410
CHCH	–22.570	0.713	0.621
CH_2CH_2	–31.669	1.350	0.678
CH_3CH_3	–40.260	1.982	0.708

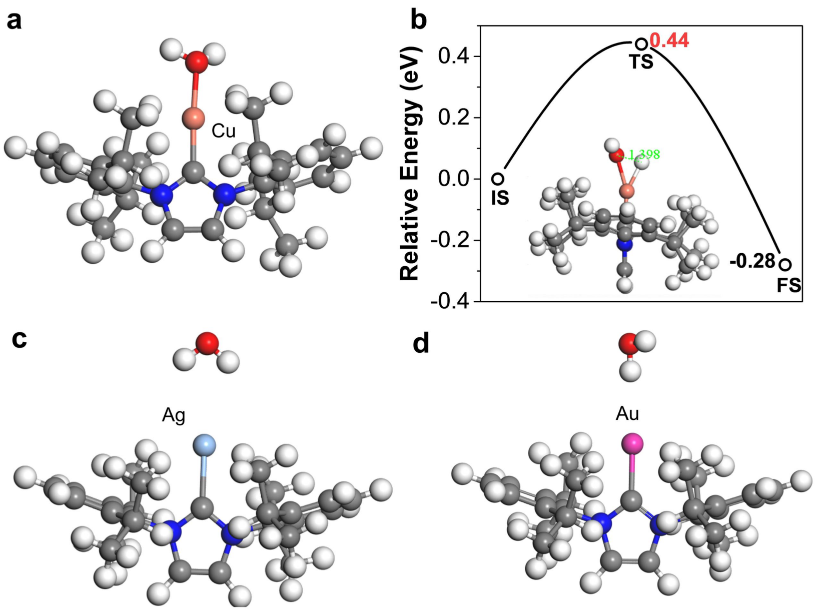

Supplementary Figure 3 | a–b, The detailed kinetic energy profile of water dissociation on NHC–Cu complex, and **c–d,** the structures of transition state for NHC–Ag and NHC–Au complex are also given. Water dissociation of NHC–Cu only has a small energy barrier of 0.44 eV, implying the pre–adsorbed H from the protons in solution or water molecule. However, water cannot form stable adsorption on NHC–Ag and NHC–Au catalysts, indicating a potentially lower ability in water dissociation and providing protons.

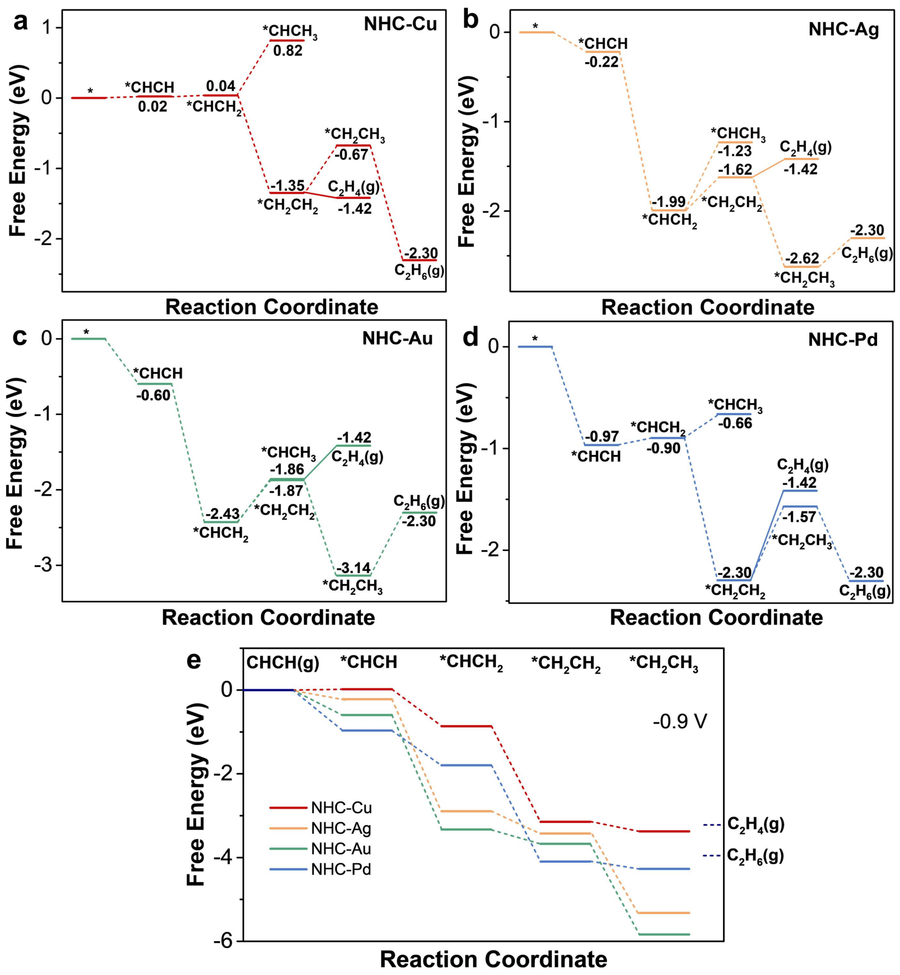

Supplementary Figure 4 | Detailed free energy diagrams of acetylene hydrogenation into C_2H_4 and C_2H_6 at **a–d,** 0 V and **e,** –0.9V vs. RHE on the NHC–Cu, NHC–Ag, NHC–Au, NHC–Pd complexes.

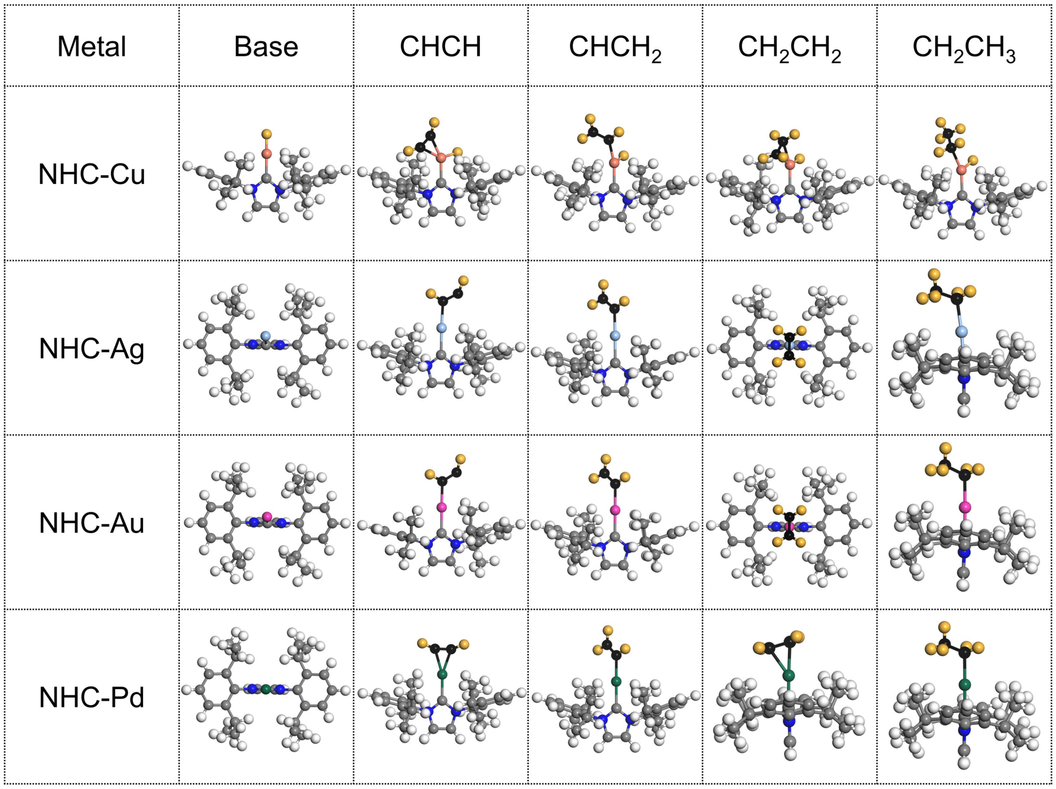

Supplementary Figure 5 | The detailed atomic structures of relevant reaction intermediates on the NHC–metal complexes. The C, N and H atoms in NHC–metal complexes are denoted by gray, blue and white balls, respectively. For comparison, the C and H atoms in CHCH hydrogenation process are denoted by black and yellow balls, respectively.

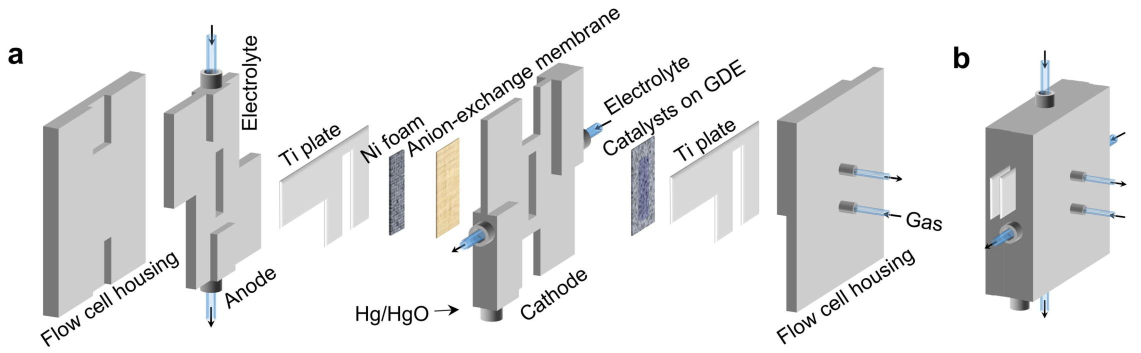

Supplementary Figure 6 | a, Exploded diagram of components in the flow cell architecture. **b,** Schematic image of a flow cell for the EAH.

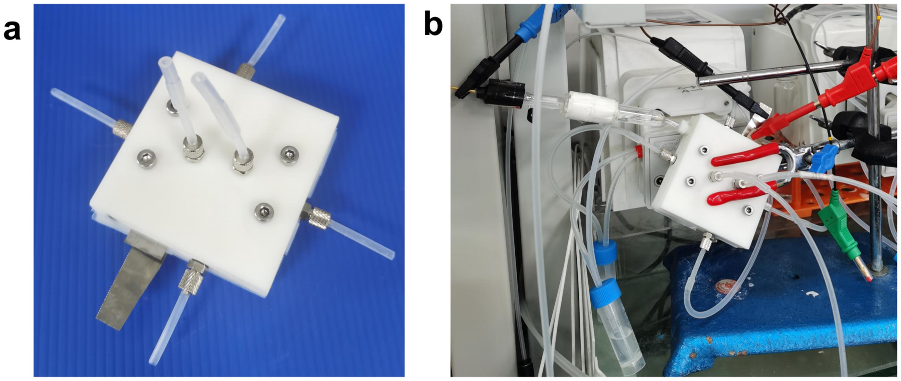

Supplementary Figure 7 | Digital images of the as–designed three–electrode flow cell with an electrode area of 1 cm^2.

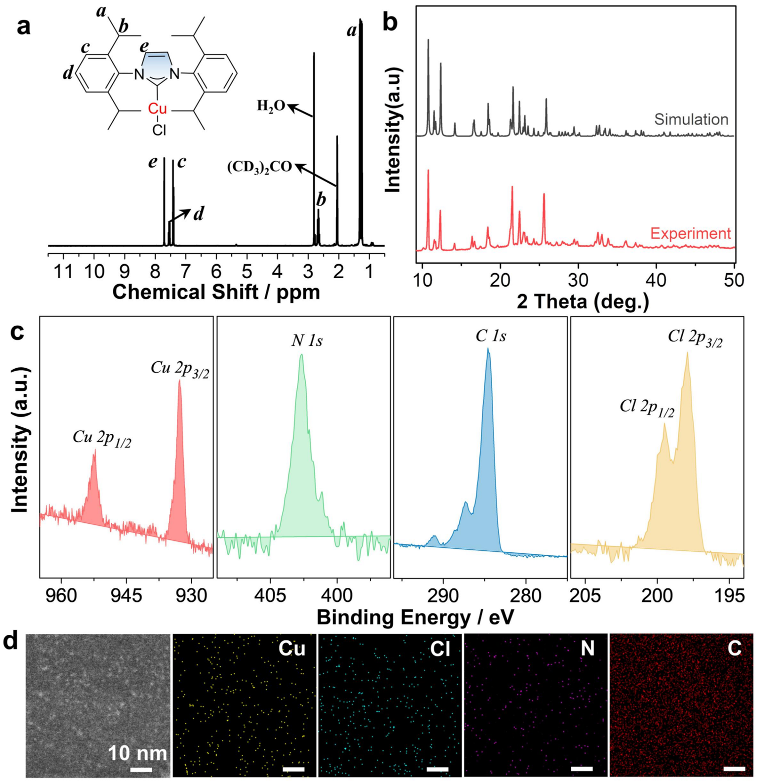

Supplementary Figure 8 | a – c, ^{1}H NMR spectra, XRD patterns and XPS spectra of NHC–Cu. **d,** HAADF–STEM images and elemental mapping images of NHC–Cu.

Notes: the chemical structure of NHC–Cu was ascertained by ^{1}H nuclear magnetic resonance (^{1}H NMR) in deuterated acetone (Supplementary Fig. 8a), including the aromatic protons (*c*, *c'*, *d*,) at 7.4–7.6 ppm, vinyl protons (*e*, *e'*) of imidazole at 7.7 ppm, methylene protons (*b* at 2.67 ppm) and methyl protons (*a* at 1.27 ppm) in isopropyl group. Those characteristic NMR peaks were in accordance with previous reports,[1-3] guaranteeing the valid structure as drawn in Supplementary Fig. 8a. X–ray diffraction (XRD) pattern in Supplementary Fig. 8b further confirmed the structure of NHC–Cu according to the simulated pattern from Cambridge Crystallographic Data Centre (CCDC, 234224). Furthermore, NHC–Cu was subjected to X–ray photoelectron spectroscopy (XPS) analysis (Supplementary Fig. 8c), containing Cu, N, C, Cl element. The peak of *Cu 2p$_{3/2}$* spectra for NHC–Cu was located at 932.6 eV without strong satellite, indicating that Cu species in NHC–Cu is Cu^{+} rather than Cu^{2+}.[4] The high–angle annular dark–field scanning transmission electron microscopy (HAADF–STEM) and corresponding

mapping results (Supplementary Fig. 8d) verified the homogeneous distribution of Cu, Cl, N and C element in NHC–Cu.

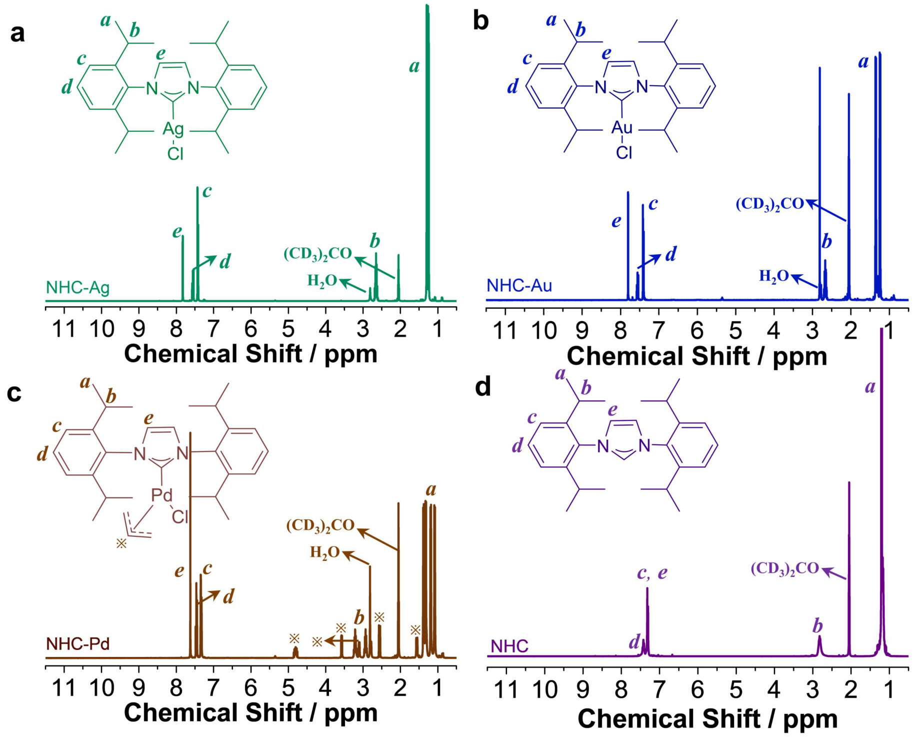

Supplementary Figure 9 | ^{1}H NMR spectra of **a,** NHC–Ag, **b,** NHC–Au, **c,** NHC–Pd and **d,** NHC in deuterated acetone. The assignment of NMR peaks was labeled in all spectra.

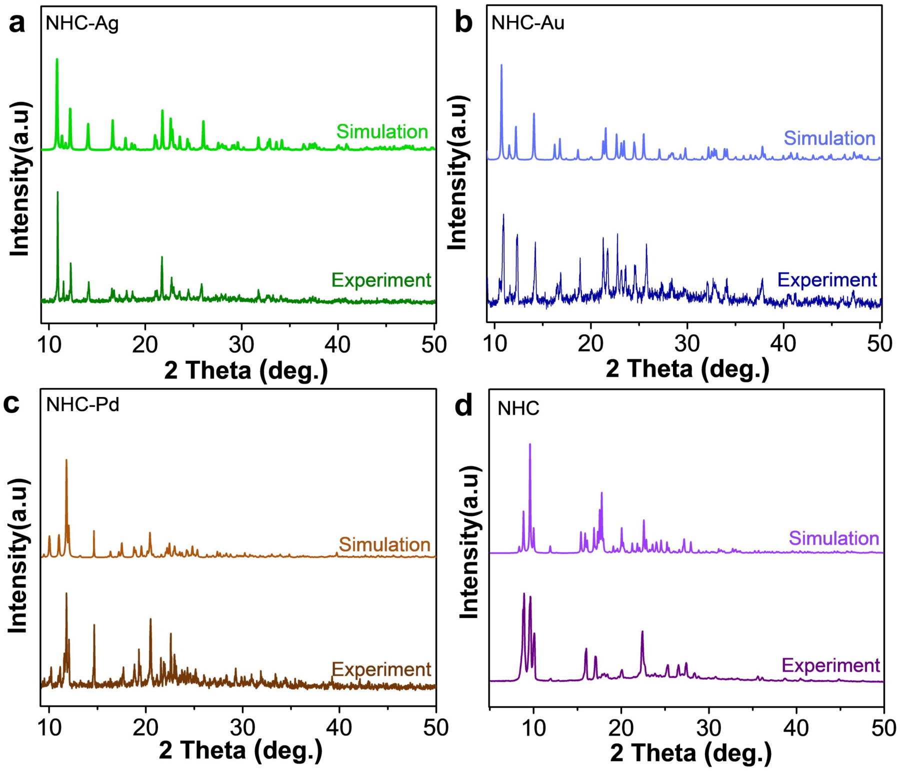

Supplementary Figure 10 | XRD patterns of **a,** NHC–Ag, **b,** NHC–Au, **c,** NHC–Pd and **d,** NHC.

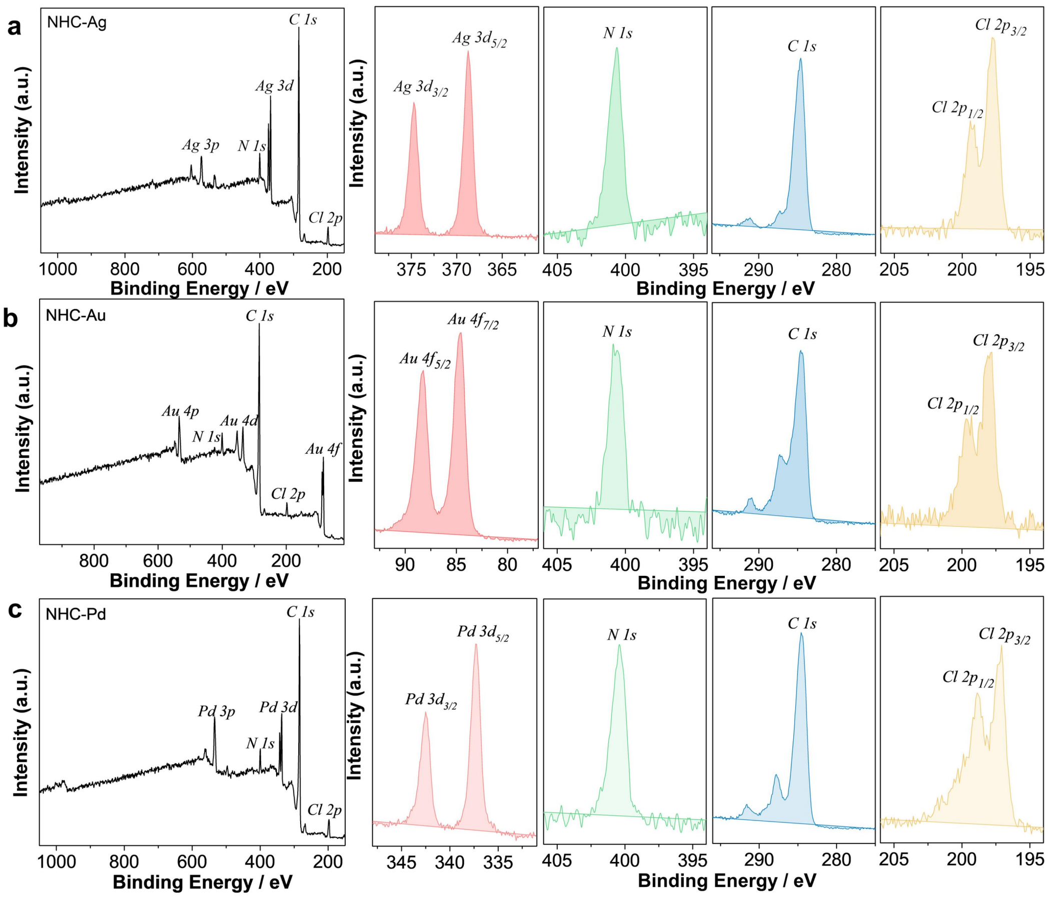

Supplementary Figure 11 | Survey XPS spectra, high–resolution *N 1s* spectra, *C 1s* spectra, *Cl 2p* spectra and metal spectra of **a,** NHC–Ag, **b,** NHC–Au and **c,** NHC–Pd.

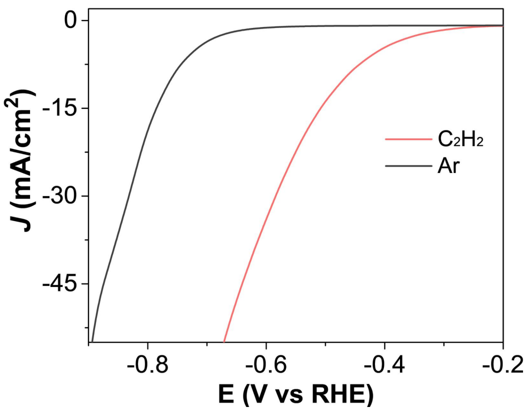

Supplementary Figure 12 | LSV curves of NHC–Cu in 1 M KOH aqueous solution under the flow of pure acetylene (red line) and argon (black line) at a scan rate of 1 mV s^{-1}.

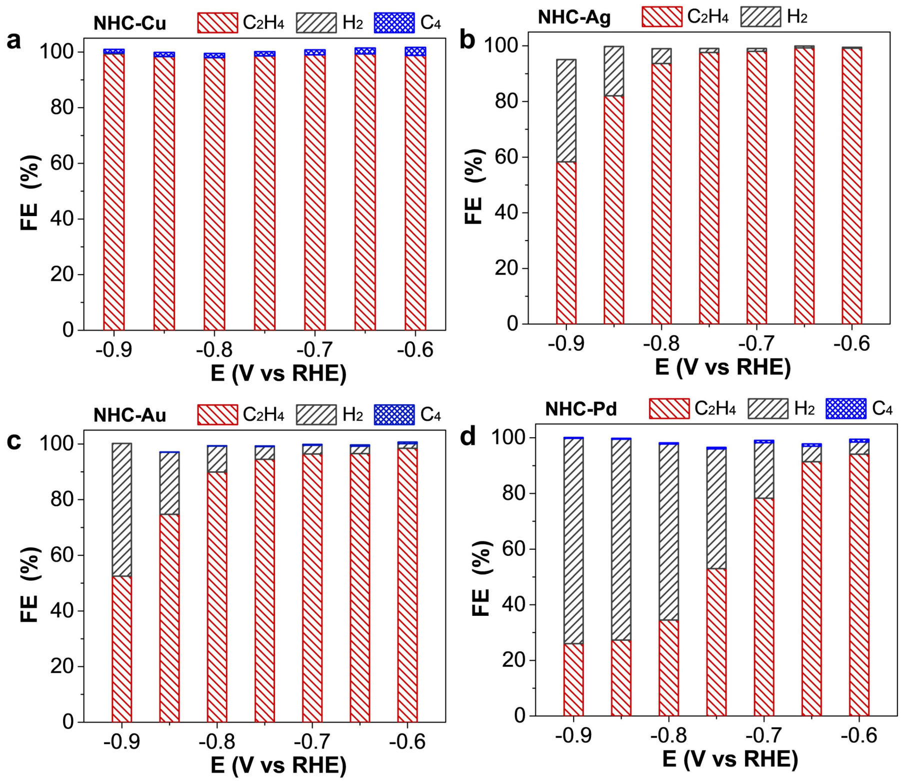

Supplementary Figure 13 | FEs of gaseous products in 1 M KOH aqueous solution under flow of pure acetylene by using **a,** NHC–Cu, **b,** NHC–Ag, **c,** NHC–Au and **d,** NHC–Pd as electrocatalysts.

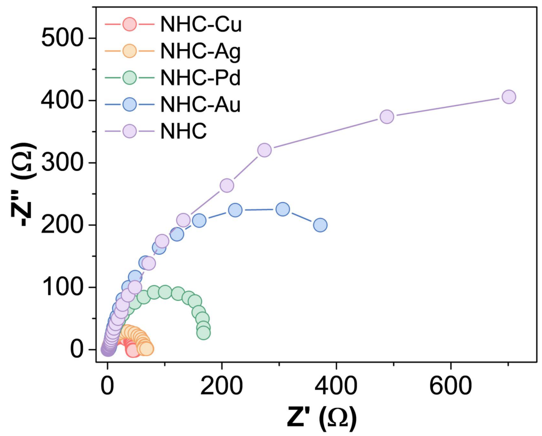

Supplementary Figure 14 | Electrochemical impedance spectroscopy (EIS) plots of NHC–Cu, NHC–Ag, NHC–Pd, NHC–Au and NHC. The electrochemical impedance spectroscopy (EIS) of different catalysts was conducted in 1 M KOH aqueous solution under the flow of pure acetylene at –0.4 V with a frequency range of 10 kHz to 0.01 Hz.

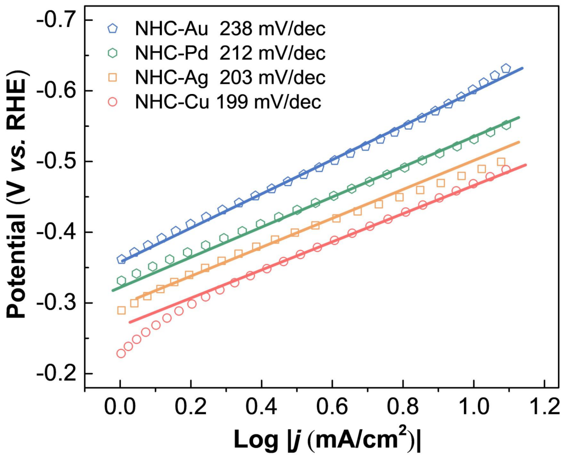

Supplementary Figure 15 | The Tafel slopes of NHC–Cu, NHC–Ag, NHC–Pd and NHC–Au. This result indicates an accelerated reaction rate of NHC–Cu for electrocatalytic acetylene semihydrogenation.

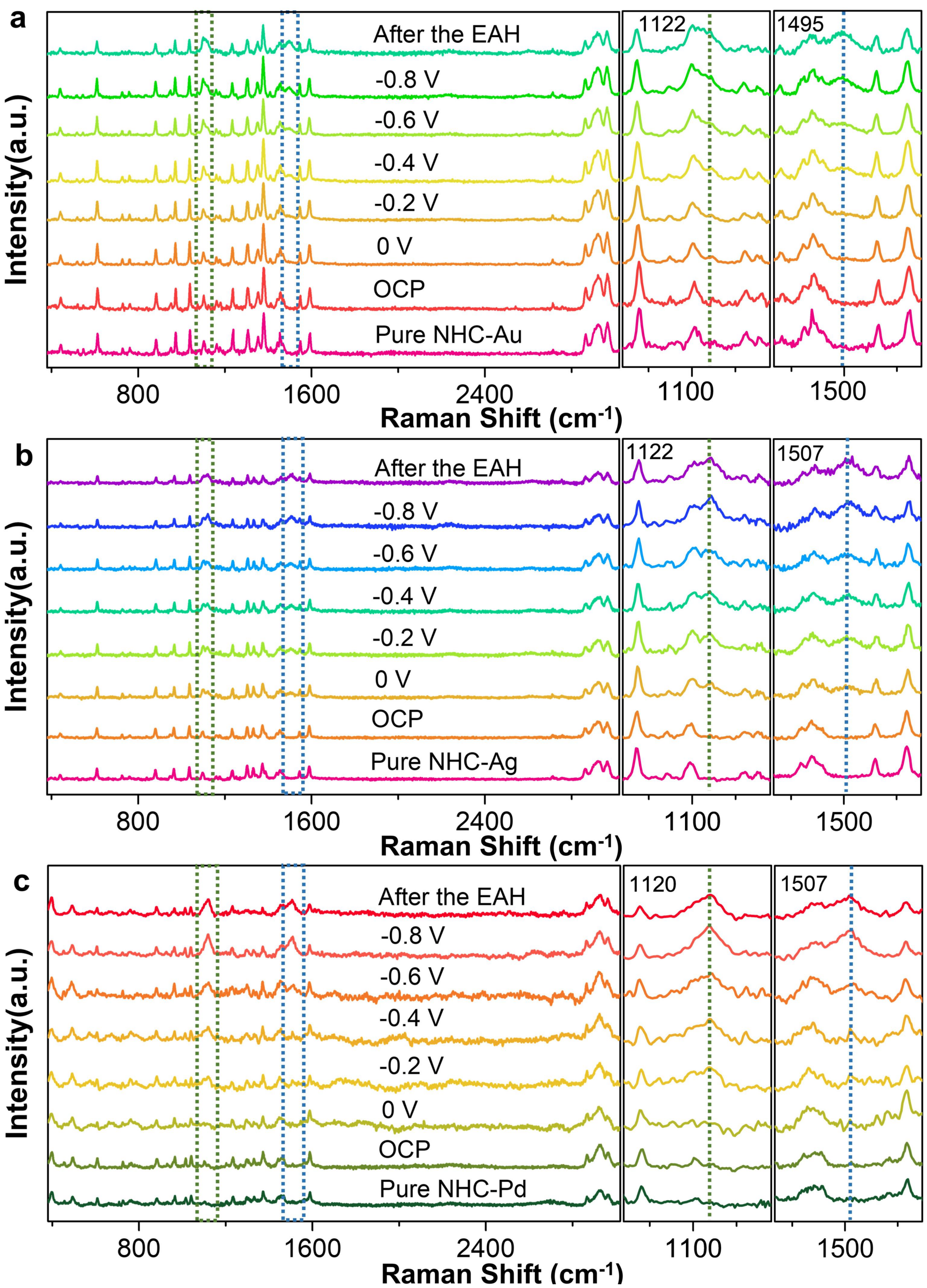

Supplementary Figure 16 | In–situ electrochemical Raman spectra of (a) NHC–Au, (b) NHC–Ag and (c) NHC–Pd in a 1 M KOH aqueous solution. For clarity, the spectral regions of 1020–1190 cm^{-1} and 1400–1610 cm^{-1} were expanded.

Notes: When the potential of NHC–Au reached at – 0.2 V, two characteristic peaks appeared at 1122 and 1495 cm^{-1} (Supplementary Fig. 16a), which were assigned to symmetric CH_2 scissors and C=C stretch modes of adsorbed ethylene, respectively.[5]

Similarly, the peaks of adsorbed ethylene on NHC–Ag appeared gradually at 1122 and 1507 cm^{-1} when the potential was increased from 0 V to –0.8 V (Supplementary Fig. 16b). For NHC–Pd, adsorbed ethylene peaks of 1120 and 1507 cm^{-1} was observed once the potential reached −0.2 V (Supplementary Fig. 16c). Here, all C=C stretch modes of NHC–Au, NHC–Ag and NHC–Pd displayed a negative shift versus 1547 cm^{-1} for NHC–Cu. Moreover, the characteristic peaks of ethylene still exist for NHC–Au, NHC–Ag and NHC–Pd when the EAH was terminated. These results unambiguously prove the weak absorption of ethylene on NHC–Cu relative to other catalysts,[5] which is consistent with theoretical results.

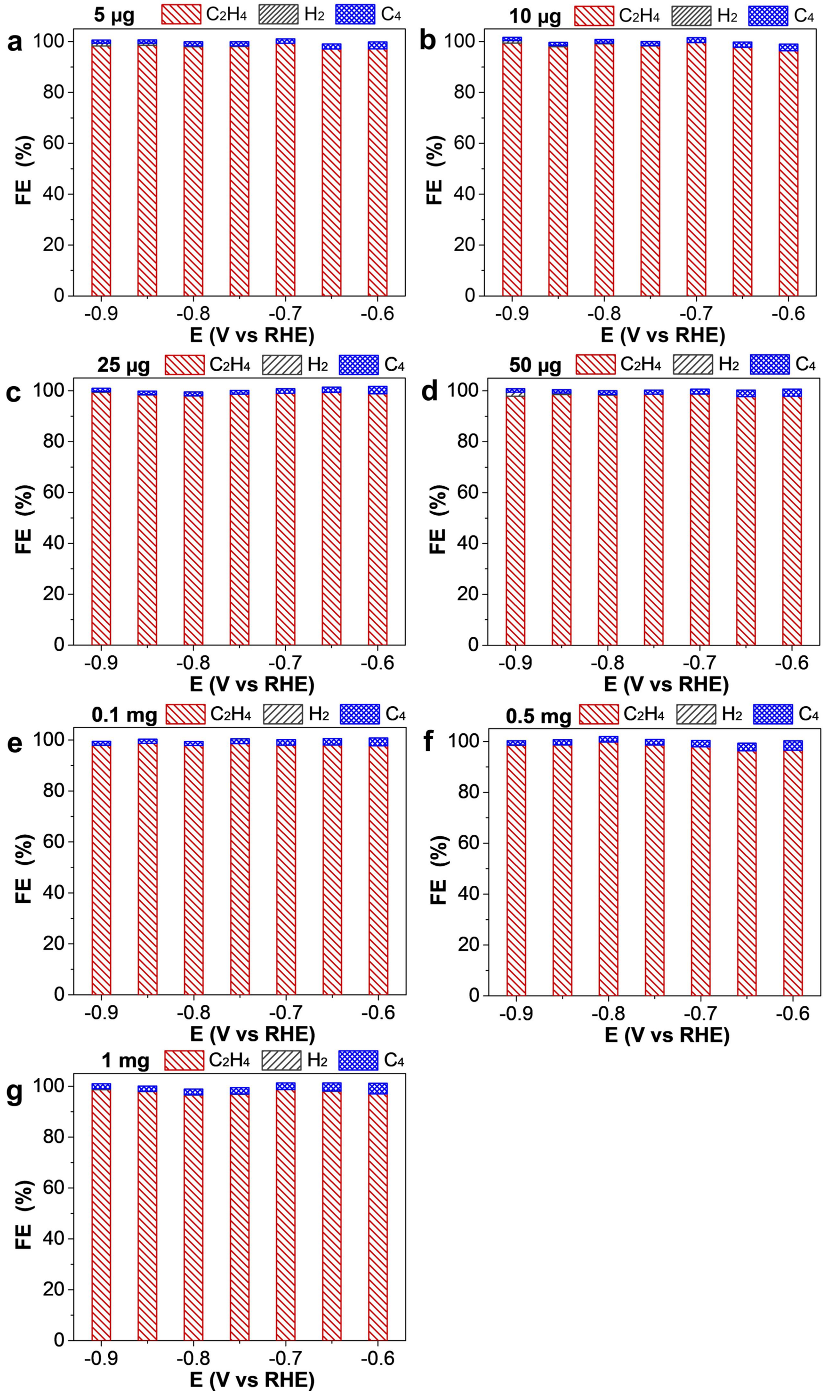

Supplementary Figure 17 | FEs of reaction products in 1 M KOH aqueous solution under the flow of pure acetylene NHC–Cu with different loading weight: **a,** 5 µg, **b,** 10 µg, **c,** 25 µg, **d,** 50 µg, **e,** 0.1 mg, **f,** 0.5 mg and **g,** 1 mg.

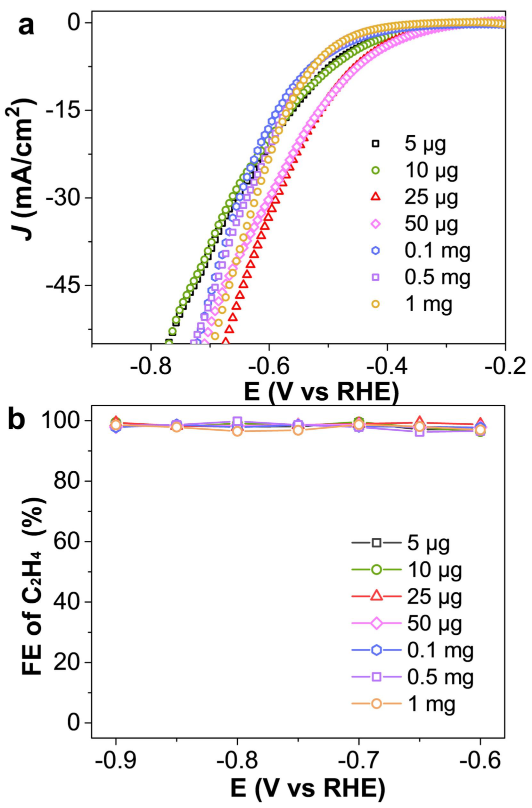

Supplementary Figure 18 | a, LSV curves of NHC–Cu with different loading weight measured in 1 M KOH aqueous solution under flow of pure acetylene at a scan rate of 1 mV s^{-1}. **b,** $FE_{ethylnene}$ of NHC–Cu with different loading weight in 1 M KOH aqueous solution under the flow of pure acetylene at different potentials. Supplementary Figs. 15–16 displayed that the optimal loading weight of NHC–Cu was 25 μg.

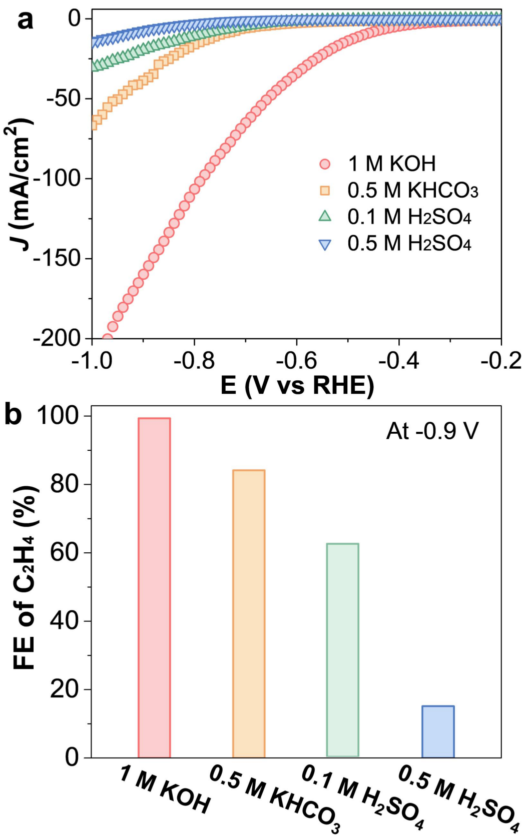

Supplementary Figure 19 | a, LSV curves of NHC–Cu in different electrolytes under the flow of pure acetylene at a scan rate of 1 mV s^{-1}. **b,** $FE_{ethylene}$ of NHC–Cu in different electrolytes under the flow of pure acetylene at –0.9 V.

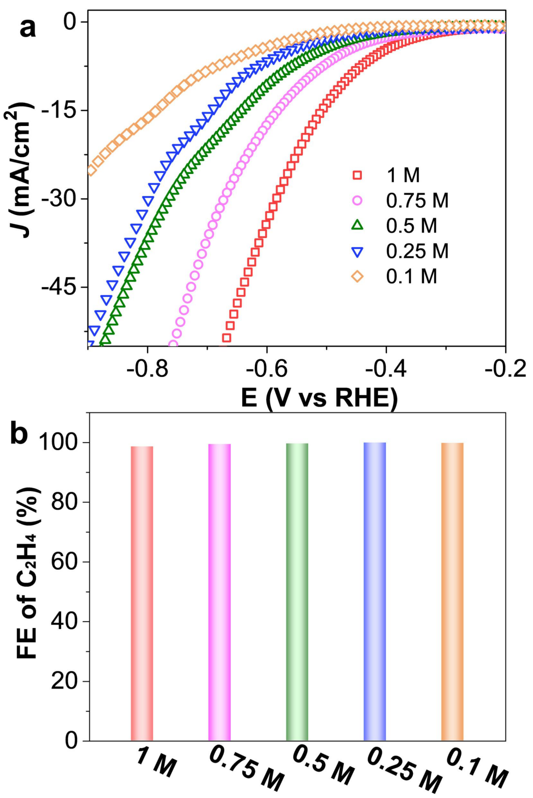

Supplementary Figure 20 | a, LSV curves of NHC–Cu in different concentrations of KOH aqueous solutions under the flow of pure acetylene at a scan rate of 1 mV s^{-1}. **b,** $FE_{ethylene}$ of NHC–Cu in different concentrations of KOH under flow of pure acetylene at –0.9 V. Supplementary Fig. S19–20 showed that the optimal electrolyte was 1 M KOH.

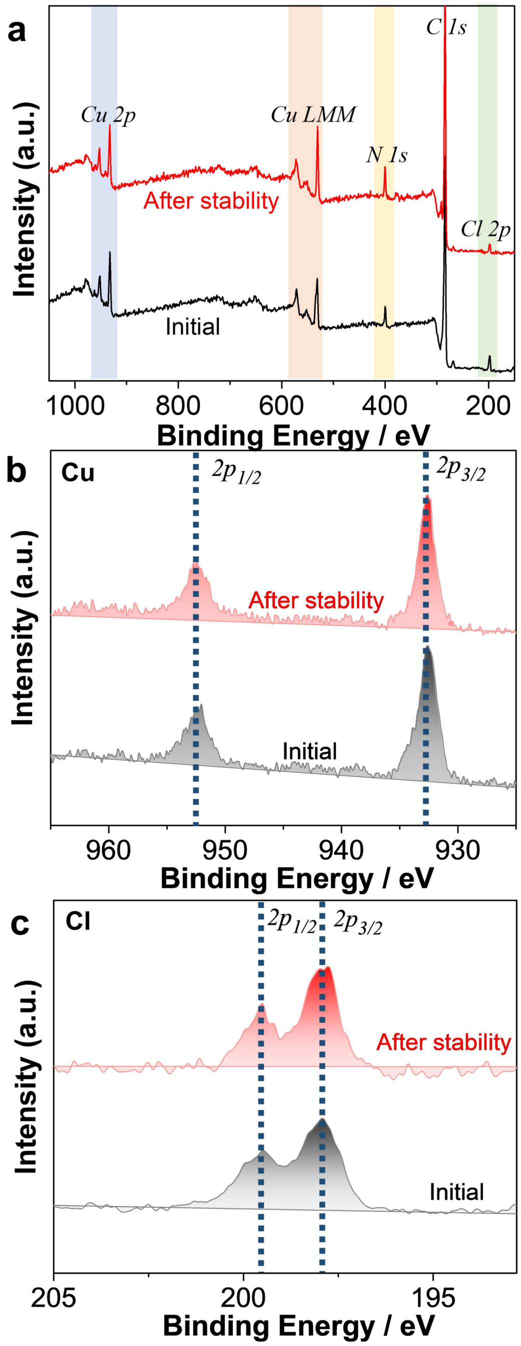

Supplementary Figure 21 | a, survey XPS spectra, **b,** *Cu 2p* spectra and **c,** *Cl 2p* spectra of NHC–Cu before and after long–term EAH stability test.

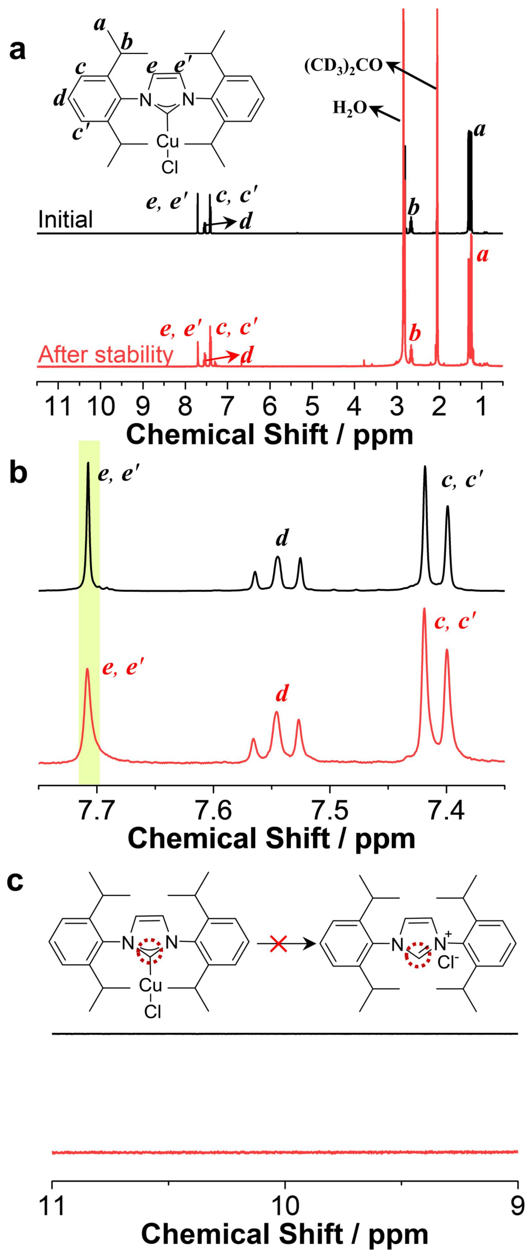

Supplementary Figure 22 | a–c, ^{1}H NMR spectra of NHC–Cu in deuterated acetone before and after long–term EAH stability test at 30 mA/cm^2.

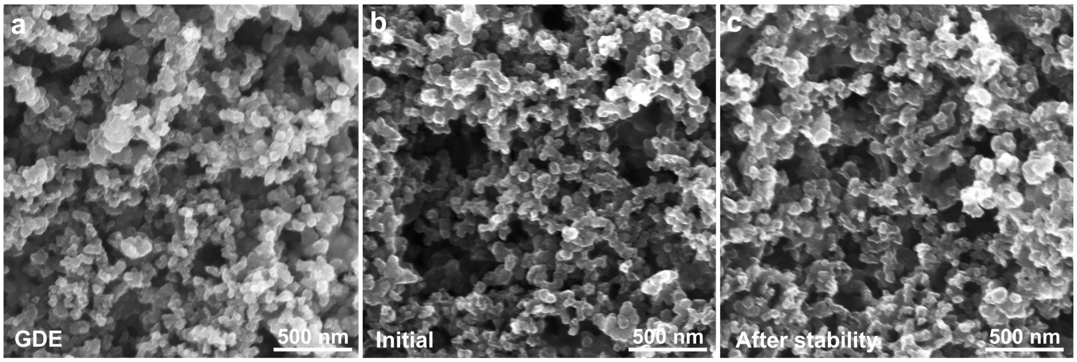

Supplementary Figure 23 | a, SEM image of gas diffusion electrode (GDE). SEM images of NHC–Cu **b,** before and **c,** after long–term EAH at 30 mA/cm^2.

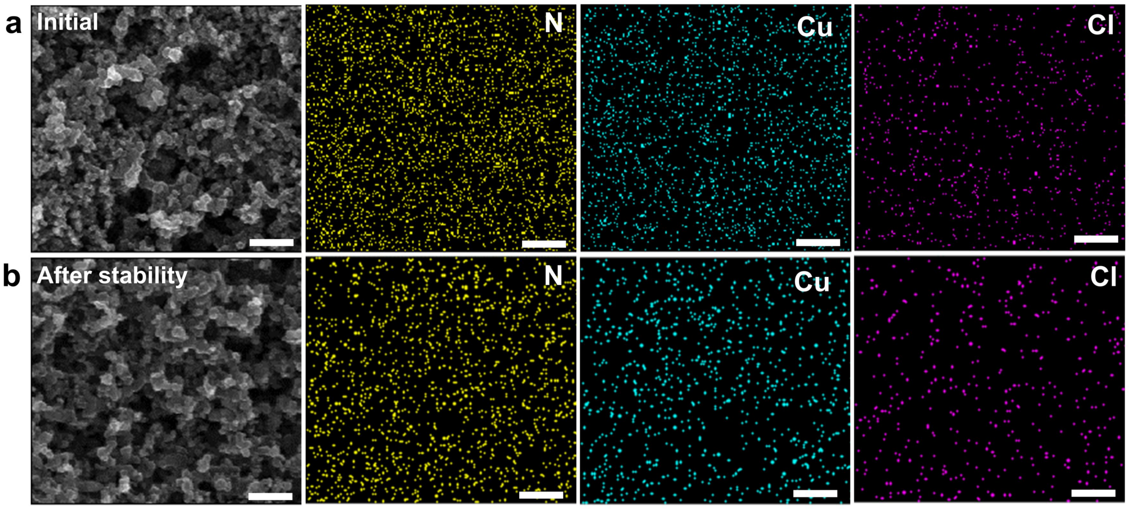

Supplementary Figure 24 | SEM and corresponding mapping images of NHC–Cu. **a,** before and **b,** after long–term EAH stability test at 30 mA/cm^2. The scale bars are 500 nm.

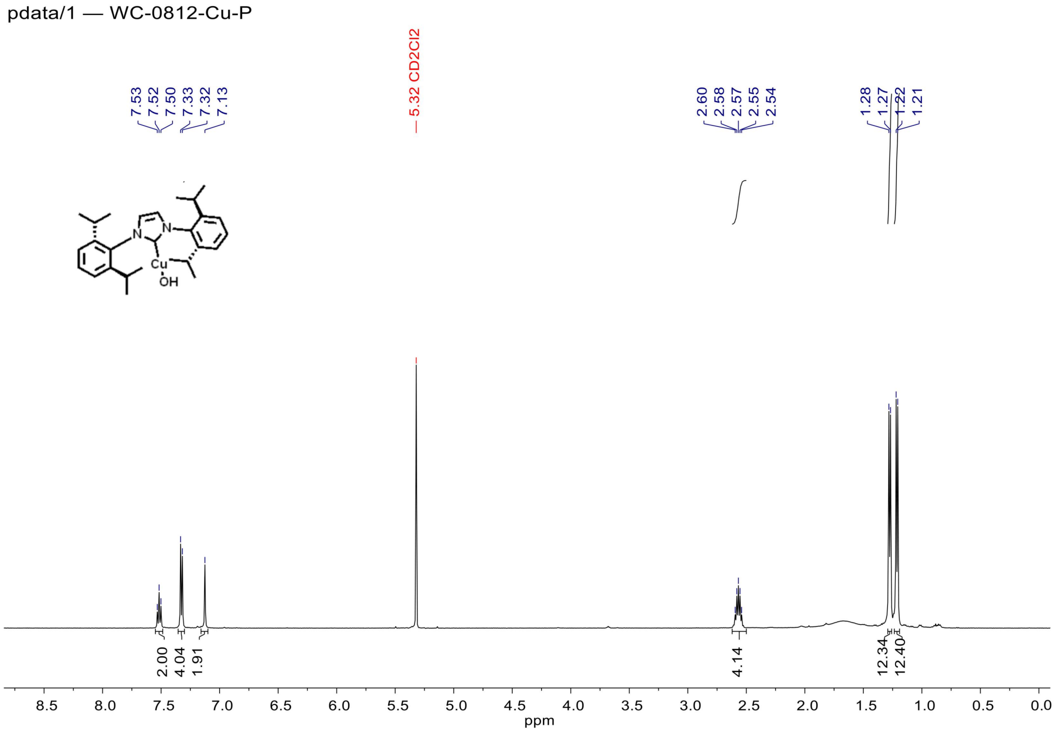

Supplementary Figure 25 | ^{1}H–NMR spectrum of NHC–Cu(OH) in deuterated dichloromethane.

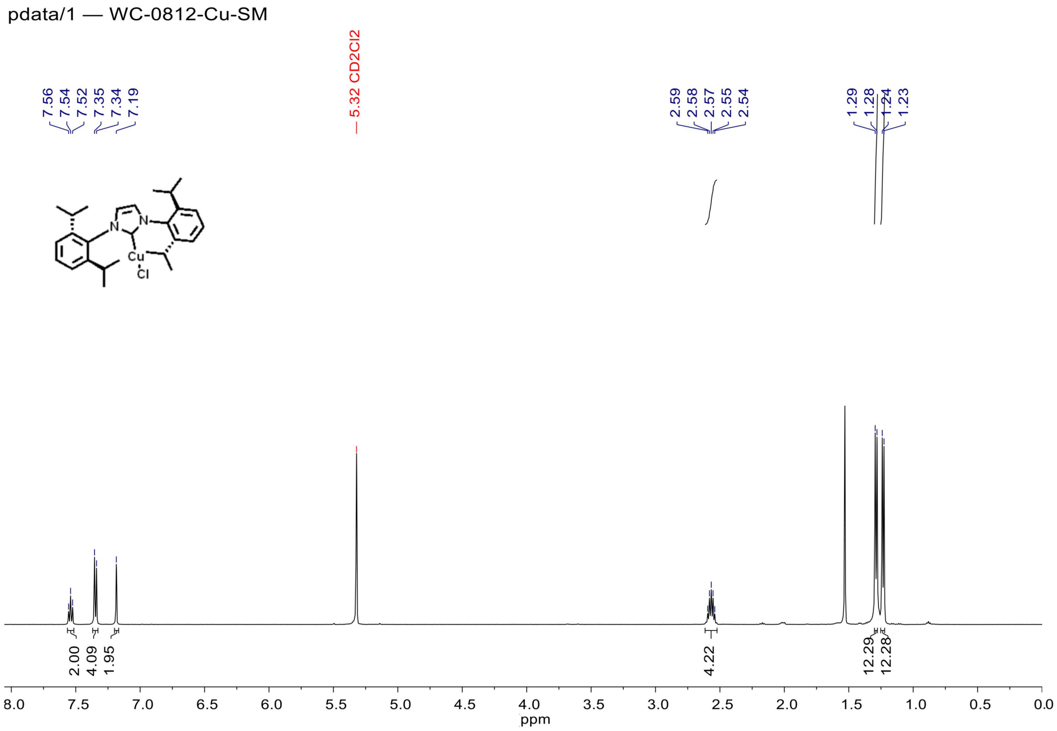

Supplementary Figure 26 | ^{1}H–NMR spectrum of NHC–Cu in deuterated dichloromethane.

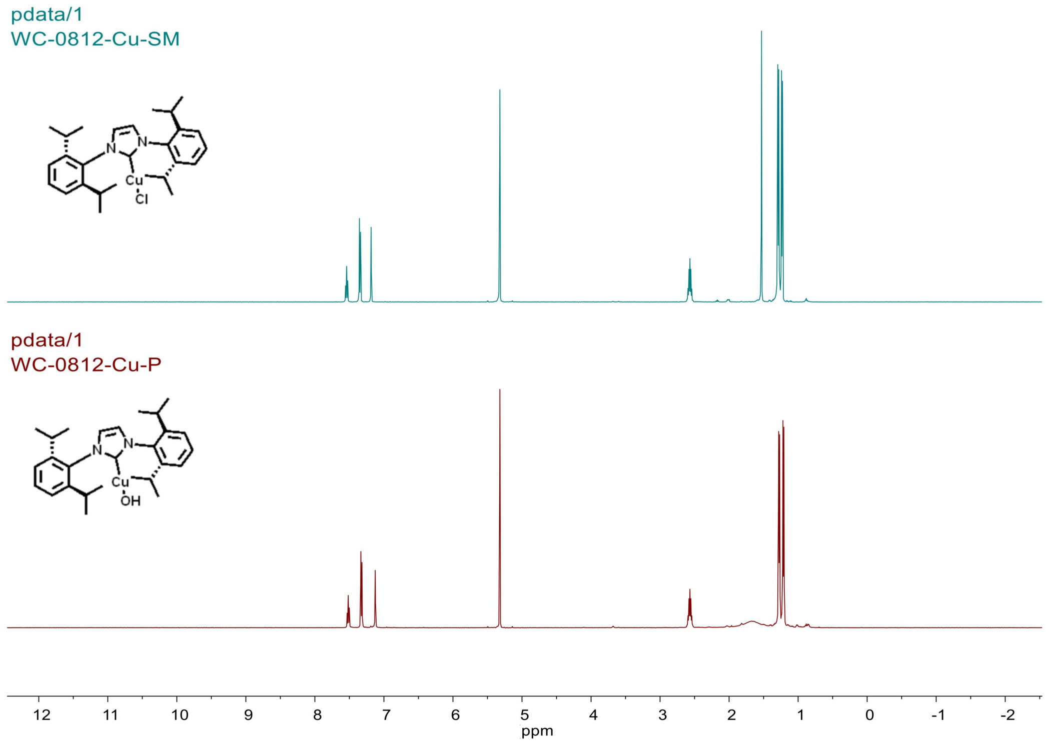

Supplementary Figure 27 | The comparison of ^{1}H–NMR spectrum of NHC–Cu and NHC–Cu(OH) in deuterated dichloromethane.

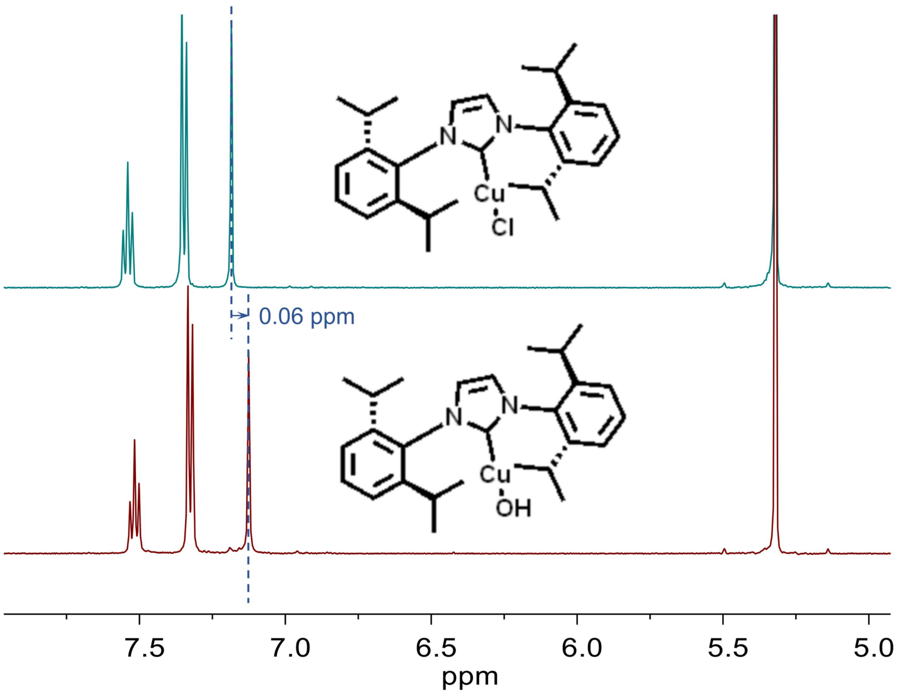

Supplementary Figure 28 | The aromatic region of ^{1}H–NMR spectrum of NHC–Cu and NHC–Cu(OH) in deuterated dichloromethane.

Notes: The ^{1}H–NMR spectrum of NHC–Cu possessed protons of imid–C*H* at 7.19 ppm in the carbene backbone. Followed by the formation of NHC–Cu(OH), the peak of imid–C*H* at 7.19 ppm shifted to a lower field (7.13 ppm, Supplementary Fig. 25–28), which was consistent with the difference of chemical shifts (0.05–0.06 ppm) between Cu – Cl and Cu–OH species.[2, 6]

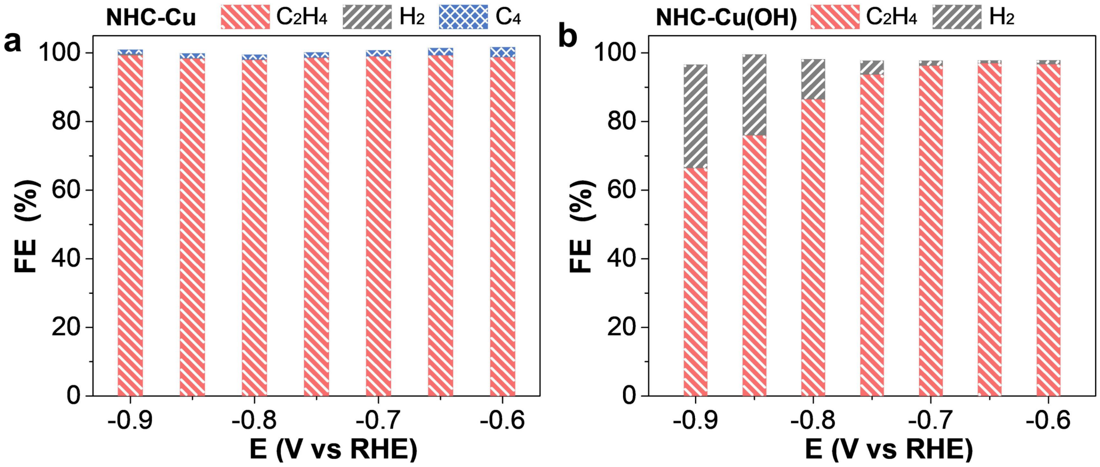

Supplementary Figure 29 | FEs of gaseous products in 1 M KOH aqueous solution under the flow of pure acetylene by using **a,** NHC–Cu and **b,** NHC–Cu(OH) as electrocatalysts.

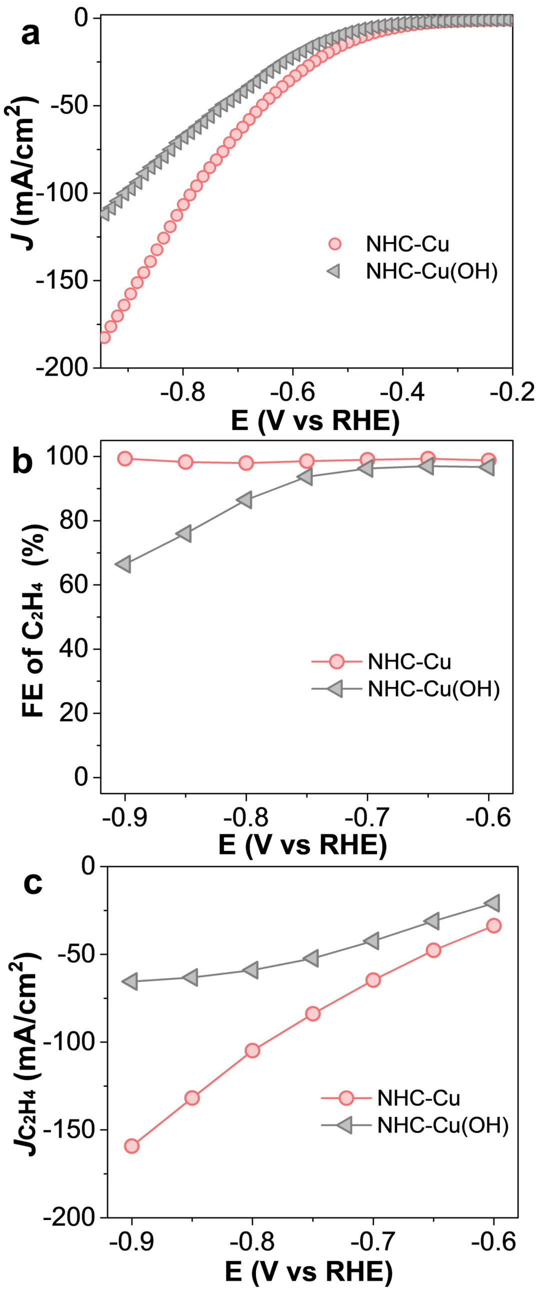

Supplementary Figure 30 | a, Polarization curves of NHC–Cu complexes (NHC–Cu and NHC–Cu(OH)) in a 1 M KOH aqueous solution under pure acetylene flow. **b,** Ethylene FEs of NHC–Cu complexes in a 1 M KOH aqueous solution at different potentials under pure acetylene flow. **c,** Partial current density of ethylene for NHC–Cu complexes in a 1 M KOH aqueous solution at different potentials under pure acetylene flow.

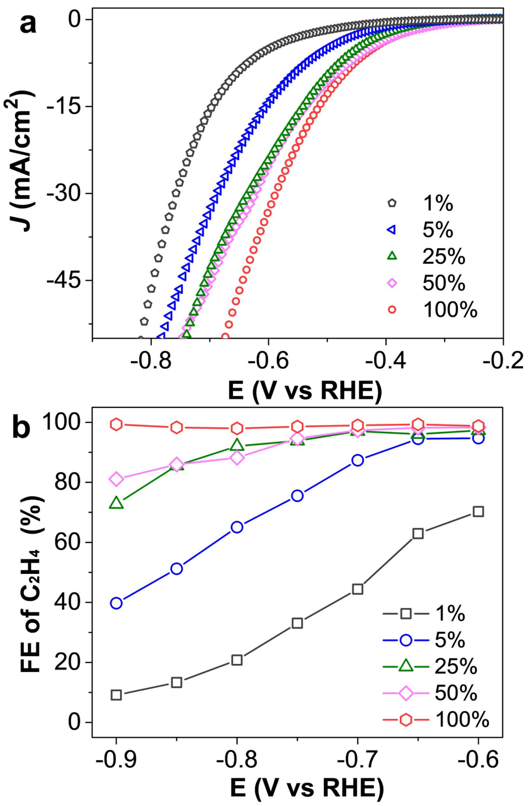

Supplementary Figure 31 | a, LSV curves of NHC–Cu in 1 M KOH aqueous solution under flow of different acetylene concentrations at a scan rate of 1 mV s^{-1}. **b,** $FE_{ethylene}$ of NHC–Cu at different potentials in 1 M KOH aqueous solution under flow of different acetylene concentrations.

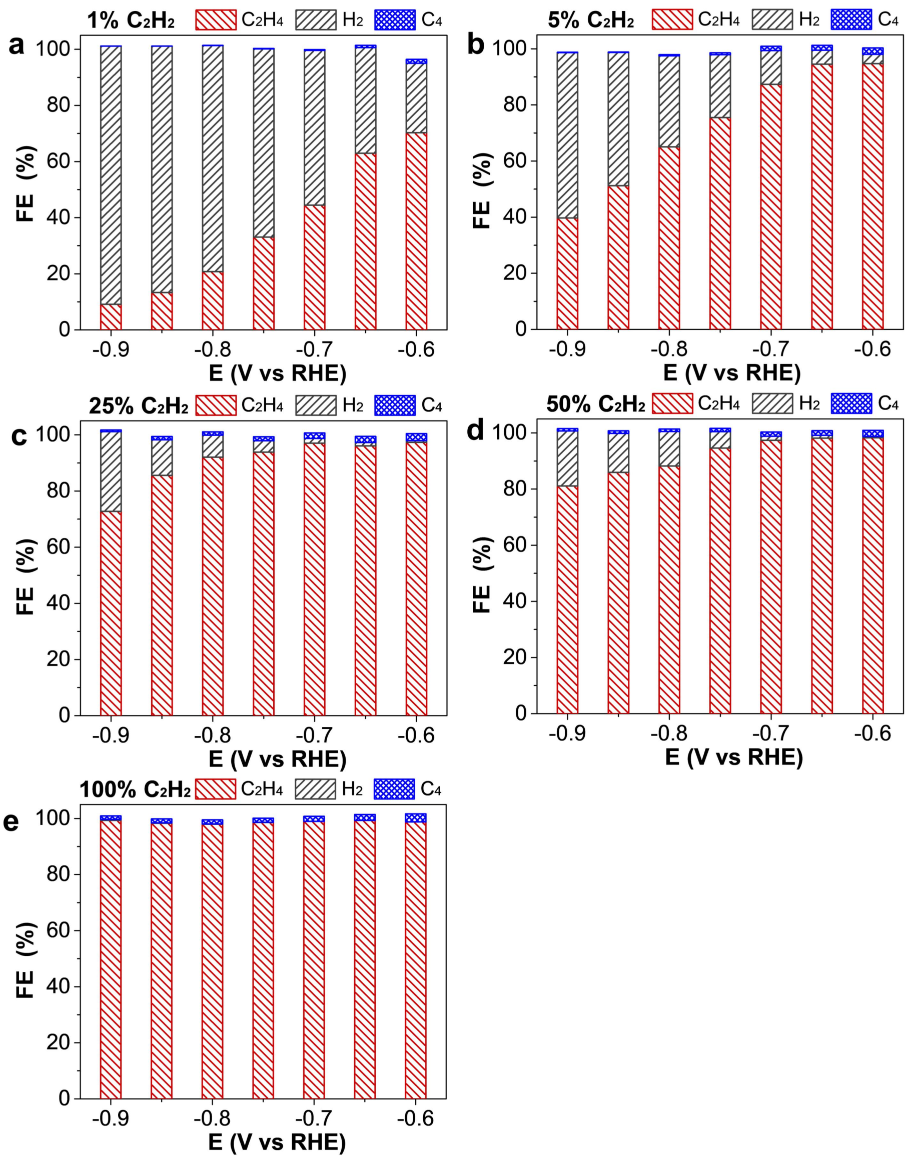

Supplementary Figure 32 | FEs distributions of NHC–Cu at different potentials in 1 M KOH aqueous solution under flow of **a,** 1 % acetylene, **b,** 5 % acetylene, **c,** 25 % acetylene, **d,** 50 % acetylene and **e,** 100 % acetylene.

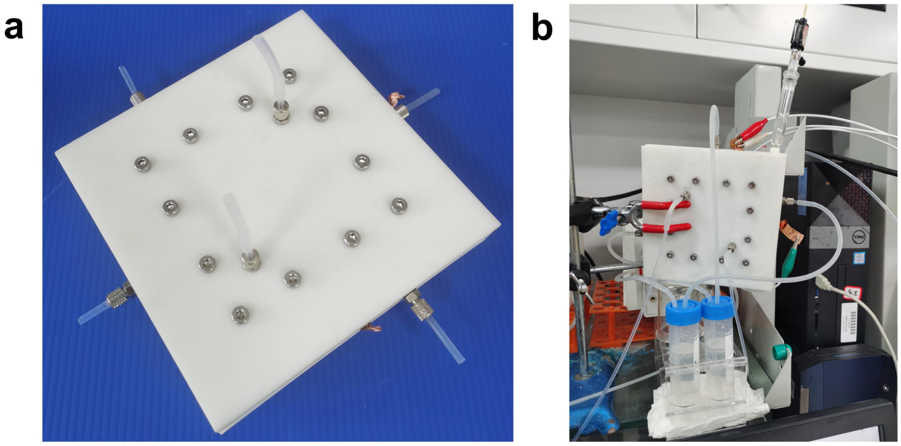

Supplementary Figure 33 | a–b, Digital images of the as–designed flow cell with an electrode area of 25 cm^2.

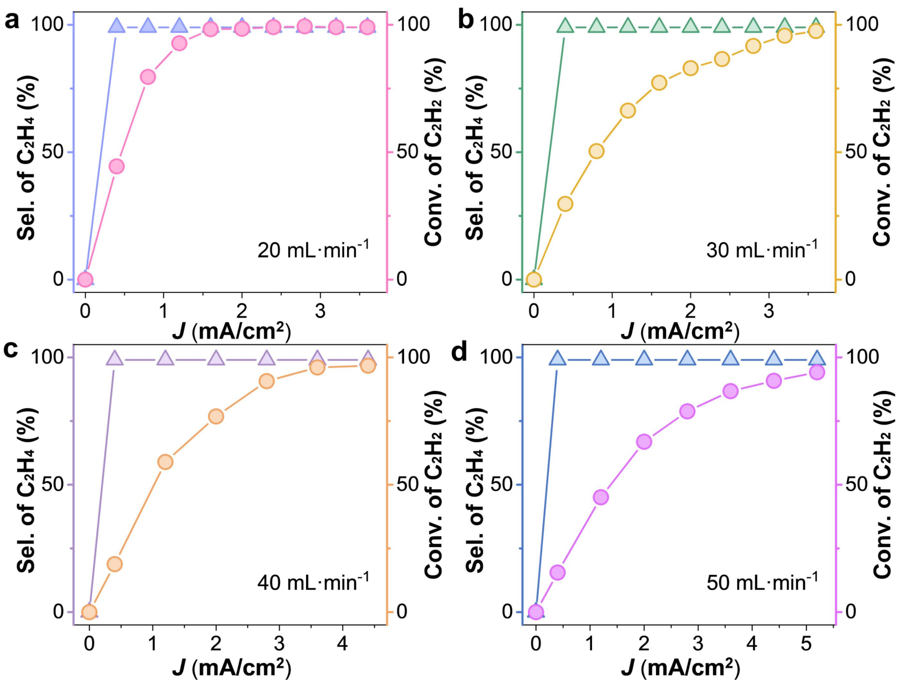

Supplementary Figure 34 | The conversion and specific selectivity of acetylene versus cathodic current in 1 M KOH aqueous solution using NHC–Cu at different flow rates of crude ethylene containing 1 % acetylene: **a,** 20 mL·min^{-1}, **b,** 30 mL·min^{-1}, **c,** 40 mL·min^{-1} and **d,** 50 mL·min^{-1}.

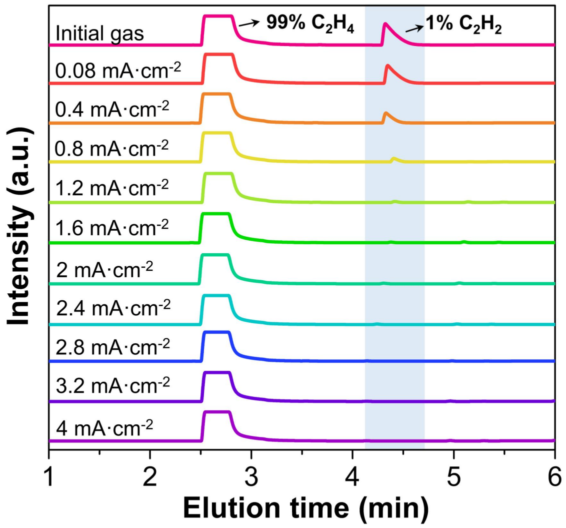

Supplementary Figure 35 | Gas product analyses from gas chromatography using NHC–Cu at different current densities for EAH under flow of crude ethylene containing 1 % acetylene.

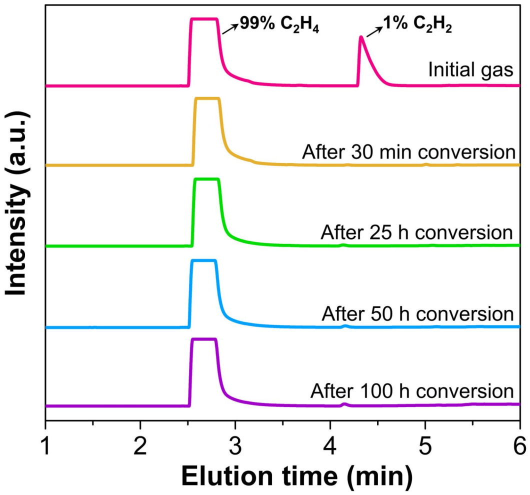

Supplementary Figure 36 | Gas product analyses from gas chromatography using NHC–Cu at 4 mA/cm^2 during different EAH stages under flow of crude ethylene containing 1 % acetylene.

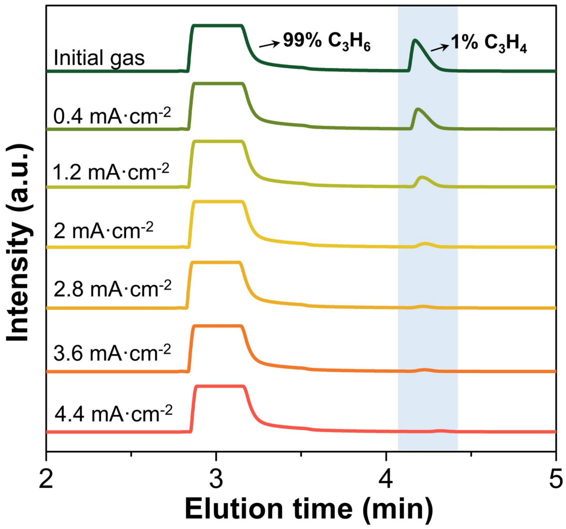

Supplementary Figure 37 | Gas product analyses from gas chromatography using NHC–Cu at different current densities for electrocatalytic propylene hydrogenation under flow of crude ethylene containing 1 % acetylene.

References

1. Liu B, Ma X, Wu F, Chen W. Simple synthesis of neutral and cationic Cu-NHC complexes. *Dalton Trans.* **44**, 1836-1844 (2015).
2. Santoro O, Collado A, Slawin AMZ, Nolan SP, Cazin CSJ. A general synthetic route to [Cu(X)(NHC)] (NHC = N-heterocyclic carbene, X = Cl, Br, I) complexes. *Chem. Commun.* **49**, 10483-10485 (2013).
3. Beillard A, Métro T-X, Bantreil X, Martinez J, Lamaty F. Cu(0), O_2 and mechanical forces: a saving combination for efficient production of Cu–NHC complexes. *Chem. Sci.* **8**, 1086-1089 (2017).
4. Yang Y, Rioux RM. Highly stereoselective anti-Markovnikov hydrothiolation of alkynes and electron-deficient alkenes by a supported Cu-NHC complex. *Green Chem.* **16**, 3916-3925 (2014).
5. Mrozek MF, Weaver MJ. Periodic trends in electrode-chemisorbate bonding: ethylene on platinum-group and gold electrodes as probed by surface-enhanced Raman spectroscopy. *J. Phys. Chem. B* **105**, 8931-8937 (2001).
6. Fortman GC, Slawin AM, Nolan SP. A Versatile cuprous synthon:[Cu (IPr)(OH)](IPr= 1, 3 bis (diisopropylphenyl) imidazol-2-ylidene). *Organometallics* **29**, 3966-3972 (2010).
