# Supplementary material for: Efficient electrocatalytic acetylene semihydrogenation by electron–rich metal sites in N–heterocyclic carbene metal complexes
Source: Nat Commun. 2021 Nov 12;12:6574. doi: 10.1038/s41467-021-26853-0 (PMC8589958; doi:10.1038/s41467-021-26853-0)
Supplement: Supplementary file 3 — Description of Additional Supplementary Files [file 41467_2021_26853_MOESM3_ESM.pdf]

Description of Additional Supplementary Files

File Name: Supplementary Data 1
Description: The results of the DFT simulations for determining the structures of the catalysts
